# A retrospective review of the two-step tuberculin skin test in dialysis patients

**DOI:** 10.1186/s40697-016-0119-4

**Published:** 2016-06-07

**Authors:** Rukhsana Foster, Thomas W. Ferguson, Claudio Rigatto, Blake Lerner, Navdeep Tangri, Paul Komenda

**Affiliations:** Seven Oaks General Hospital Renal Program, 2PD12 - 2300 McPhillips Street, Winnipeg, MB R2V 3M3 Canada; Chronic Disease Innovation Centre, Seven Oaks General Hospital, Winnipeg, Canada; Department of Medicine, University of Manitoba, Winnipeg, Canada

**Keywords:** Chronic renal failure, Dialysis, End-stage renal disease, Immunosuppression, Kidney transplantation, Latent tuberculosis infection, Tuberculin skin test, Tuberculosis

## Abstract

**Background:**

Reactivation of latent *Mycobacterium tuberculosis* infection (LTBI) is a health concern for patients on dialysis or receiving a kidney transplant, as these patients are often immunosuppressed. The most frequently used test for LTBI screening in this population is the tuberculin skin test (TST). The diagnostic accuracy (sensitivity and specificity) of the TST in a contemporary North American or Western European dialysis population is unknown.

**Objectives:**

Our objective was to determine the diagnostic accuracy and clinical utility of the two-step TST in patients receiving dialysis.

**Design:**

This is a retrospective cohort study.

**Setting:**

This study is set at four tertiary dialysis units across Winnipeg, Manitoba.

**Patients:**

There are 483 chronic hemodialysis and peritoneal dialysis patients in the study.

**Measurements:**

The measurements are sensitivity and specificity of the TST with respect to abnormal chest X-ray.

**Methods:**

All patients received a two-step TST and assessment of risk factors for prior tuberculosis (TB) infection between February 2008 and December 2008. This cohort was retrospectively linked to our tuberculosis registry to ascertain if prophylaxis was received for LTBI.

**Results:**

At an induration cutoff of 5 mm, 62 (13 %) patients had a positive two-step TST. Patients with a known Bacillus Calmette-Guérin (BCG) vaccination were more likely to test positive (50 % of those with a positive TST had a BCG versus 34 % with a negative TST, *p* = 0.05). Using a diagnostic gold standard of an abnormal chest X-ray as a proxy for LTBI, the sensitivity of the TST was only 14 % and the specificity was 88 %. Only 8 of 62 patients with a positive TST (13 %) received prophylaxis for LTBI. None of the patients who tested negative were treated.

**Limitations:**

There is a lack of a truly accurate gold standard for LTBI.

**Conclusions:**

The TST has limited diagnostic and clinical utility for LTBI screening in patients on dialysis. Further research into the diagnostic accuracy of interferon-gamma release assays and a revision of LTBI screening guidelines in patients on dialysis should be considered.

**Electronic supplementary material:**

The online version of this article (doi:10.1186/s40697-016-0119-4) contains supplementary material, which is available to authorized users.

## Abrégé

**Mise en contexte:**

La réactivation d’une infection latente à *Mycobacterium Tuberculosis* demeure une préoccupation constante pour la santé des patients recevant des traitements de dialyse de même que pour les receveurs d’une greffe puisque ces patients sont souvent immunodéprimés. Le test le plus fréquemment utilisé pour le dépistage des infections tuberculeuses latentes (ITL) chez cette population est le test cutané à la tuberculine (TCT). Néanmoins, l’exactitude du diagnostic (sensibilité et spécificité) du TCT demeure inconnue pour les populations de patients actuellement sous dialyse en Amérique du nord et en Europe de l’ouest.

**Objectif de l’étude:**

Cette étude visait à évaluer la précision diagnostique et la pertinence clinique de ce test de dépistage en deux étapes chez les patients recevant des traitements de dialyse.

**Cadre et type d’étude:**

Il s’agit d’une étude de cohorte rétrospective qui s’est tenue dans quatre unités de dialyse en centre de soins tertiaires à Winnipeg, au Manitoba.

**Patients:**

La cohorte était constituée de 483 patients sous hémodialyse chronique ou sous hémodialyse péritonéale.

**Mesures:**

La sensibilité et la spécificité du TCT ont été évaluées sur la base d’une radiographie pulmonaire anormale.

**Méthodologie:**

Au cours de la période comprise entre février et décembre 2008, tous les participants avaient subi les deux étapes du TCT et fait l’objet d’une évaluation de leurs facteurs de risque attribuables à des antécédents d’infection tuberculeuse. La cohorte a été reliée de façon rétrospective aux registres actuels de la tuberculose afin de vérifier si les mesures de prophylaxie avaient été suivies.

**Résultats:**

À la valeur seuil d’induration de 5 mm, 62 patients (13 %) ont obtenu un résultat positif au TCT réalisé en deux étapes. Les patients qui avaient été vaccinés contre le BCG (bacille de Calmette et Guérin) par le passé étaient plus susceptibles d’obtenir un résultat positif. La moitié (50 %) des patients présentant un résultat positif au TCT avaient reçu le vaccin alors que cette proportion était de seulement 34 % parmi les patients qui avaient eu un résultat négatif (p = 0,05). À titre d’indicateur de la présence d’une ITL, le recours à une radiographie pulmonaire anormale a permis d’établir la sensibilité du TCT à 14 % alors que la spécificité s’est élevée à 88 %. Seuls huit des 62 patients ayant obtenu un résultat positif au TCT avaient suivi les mesures prophylactiques adéquates et aucun des patients ayant obtenu un résultat négatif au TCT n’a été traité.

**Limites de l’étude:**

Il n’existe aucune norme de référence véritablement précise en ce qui concerne les ITL.

**Conclusions:**

Le test cutané à la tuberculine s’avère d’une pertinence diagnostique et clinique limitée pour le dépistage des infections tuberculeuses latentes chez les patients sous dialyse. La fiabilité diagnostique de mesures telles que le test de libération d’interféron gamma devrait être davantage explorée. De plus, une révision des lignes directrices en matière de dépistage des ITL chez les patients sous dialyse devrait être prise en considération.

## What was known before

The two-step tuberculin skin test is often used to diagnose latent tuberculosis infection (LTBI) in dialysis patients. This test has been shown to offer poor diagnostic accuracy in this population due to their immunosuppressed state and the cross-reaction with the Bacillus Calmette-Guérin (BCG) vaccination.

## What this adds

This is one of the largest evaluations of the diagnostic accuracy of the TST in a dialysis population within a non-endemic North American population. Additionally, we followed patients to ascertain if prophylaxis was received following diagnosis.

## Background

*Mycobacterium tuberculosis* (tuberculosis (TB)) infection is a major global health concern. An estimated nine million new cases of TB were diagnosed in 2013, and up to one third of the world population is estimated to have latent tuberculosis infection (LTBI) [1, 2]. Patients with kidney failure are at particularly high risk for LTBI reactivation. These patients have altered cell-mediated immunity that manifests as systemic immunodeficiency and other risk factors for activation such as older age, immune suppressive therapy, and comorbid conditions such as diabetes [3–5]. Patients receiving dialysis are 10–25 times more likely to experience reactivation of LTBI than the general population [6, 7].

Reactivation of LTBI and its spread within the hemodialysis unit are of particular concern, as transmission in hemodialysis units is facilitated by frequent hospital visits, dialyzing in close proximity, and underlying immune dysfunction as a result of kidney failure [8, 9]. Moreover, diagnosis of TB is challenging in kidney disease because of its atypical presentation characterized by an insidious onset, symptoms mimicking those of kidney failure, and more frequent extrapulmonary manifestations [10]. Furthermore, identifying and treating LTBI prior to transplantation in transplant-eligible patients is critical because these patients are at extreme risk for reactivation post transplantation as a consequence of induction and maintenance immunosuppressive therapy [11, 12]. Currently, the international guidelines body, Kidney Disease: Improving Global Outcomes (KDIGO), advises screening of immunosuppressed kidney failure patients with the tuberculin skin test (TST) followed by the management of TST-positive patients with anti-TB prophylaxis [13, 14].

Although the TST is an accurate diagnostic tool for LTBI in the general population with a sensitivity approaching 100 % [15], there are several factors which likely diminish its utility in patients with kidney failure. Uremic immune anergy can decrease sensitivity, whereas prior exposure to non-tuberculosis mycobacteria or BCG vaccination, both more frequent in dialysis populations, can greatly reduce the specificity of the TST [7, 11, 16, 17]. Information regarding the accuracy of the TST in a North American or Western European dialysis population is lacking because most studies to date have been conducted in countries with endemic rates of TB [12]. The objective of the present study was to determine the diagnostic accuracy of the TST in detecting LTBI in hemodialysis patients, using prospective data from a Canadian provincial screening program.

## Methods

Ethics approval was obtained from the University of Manitoba Health Research Ethics Board (ethics # HS15663); the Winnipeg Regional Health Authority (WRHA) and Manitoba Health Information Privacy Committee (HIPC) provided approval for all linkages and uses of the data.

### Data sources

#### Manitoba renal program TB screening database

The Manitoba Renal Program (MRP) includes four major hospital dialysis units in Winnipeg, Manitoba. Between September 2007 and February 2008, there were four confirmed cases of active TB diagnosed within the MRP, leading to the implementation of a comprehensive screening protocol from February 2008 to December 2008. It included a two-step TST in 483 patients dialyzing in Winnipeg during that time. The screening process involved a patient questionnaire, abstraction of clinical data on risk factors from patient records, chest radiograph, and two-step TST. Following a positive test, patients were referred for follow-up and treatment if deemed appropriate by a clinician.

During screening, the following clinical data was gathered: demographic information such as age, sex, dialysis modality, race, and country of origin and risk factors for TB including previous TB infection, history of close contact with active TB cases, BCG vaccination status, human immunodeficiency virus (HIV) infection or other cause of immunosuppression, and previous TST results. The chest radiograph was examined by a radiologist for evidence of TB lesions, and the dialysis unit nursing staff administered the TST. This involved a 5-unit (0.1 mm) intradermal injection of tuberculin purified protein derivative (PPD) on the volar aspect of the patient’s arm (Mantoux method). The first injection site was examined between 48 and 72 h after administration, and if negative, a second “booster” injection was given 1 to 2 weeks later. The diameter of the induration was measured and recorded. Data was collected on paper case report forms and subsequently entered into an electronic database.

#### Manitoba TB and LTBI registries

TB is a reportable disease in Canada, and surveillance of TB in Manitoba is the responsibility of the Manitoba Health (MH)—Public Health Branch. As such, the MH is responsible for maintaining provincial registries of all laboratory and clinically confirmed cases of TB, as well as individuals confirmed as having LTBI, and those who subsequently received prophylaxis for LTBI. Accessed through the MH and the Public Health Agency of Canada, these registries were linked to the MRP screening database in order to identify individuals diagnosed with LTBI and/or prescribed pharmacotherapy for LTBI. It should be noted that Manitoba has a stable population with an emigration rate of <1.5 % [18], making these registries reliable sources for tracking patient outcomes.

#### Integrated Public Health Information System (iPHIS)

This database tracks cases of active TB infection and was used to verify which patients developed active TB as per the World Health Organization (WHO) criteria.

#### Data linkages

**Fig. 1:**
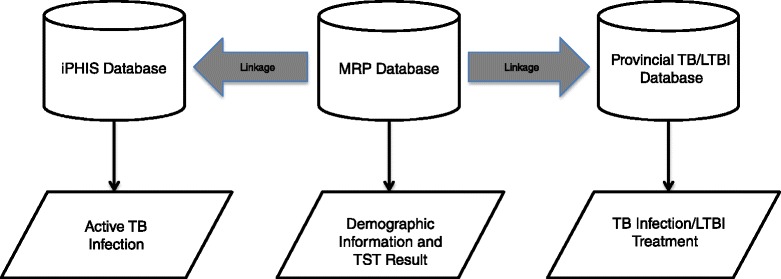
De-identified patient data linkage pathway. Legend: *iPHIS* Integrated Public Health Information System, *MRP* Manitoba Renal Program, *TB* tuberculosis, *LTBI* latent tuberculosis infection

The databases were linked via a unique patient health number. Patient data was linked by the respective holding agencies and completely de-identified prior to analysis, maintaining patient anonymity (Fig. 1).

### Statistical analysis

Patient characteristics were summarized, tabulated, and compared using appropriate tests (*t* test, chi-square test, and Fisher’s exact test where applicable). The primary aim of this study was to ascertain the accuracy of the TST in diagnosing LTBI. We used the presence of radiographic evidence of previous TB infection, including pulmonary infiltrates, tissue loss, and cavitations in the upper lobe segments, as the reference standard for diagnosis of LTBI. The chest radiograph was available and accurately reported in 96 % of the patients. Additional WHO criteria for LTBI including history of TB and close contact with active TB were not as complete, but we did use a composite including chest X-ray or common risk factors from published literature [12] in a sensitivity analysis. This approach has been widely used due to limitations in reporting and collection of data pertaining to TB risk factors [19, 20]. We classified the TST response as either positive or negative, using both 5 (primary analysis) and 10 mm (secondary analysis) induration thresholds. Two-by-two classification tables were created, and standard diagnostic test performance characteristics (sensitivity and specificity) were determined for each TST threshold.

As secondary aims, we additionally calculated the sensitivity and specificity of the TST and chest radiograph with respect to the clinical diagnosis of LTBI in the LTBI registry and determined the number of patients who received treatment for active TB over the 5-year follow-up period.

## Results

**Fig. 2:**
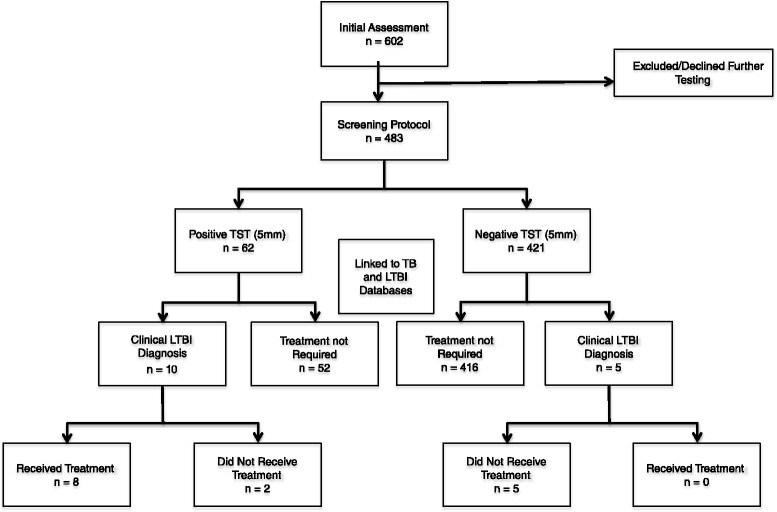
Summary from the LTBI screening protocol and data linkages. Legend: *TST* tuberculin skin test, *TB* tuberculosis, *LTBI* latent tuberculosis infection, *TB/LTBI databases*, provincial databases tracking patients diagnosed with TB or receiving LTBI prophylaxis

A total of 602 patients underwent initial risk assessment for LTBI. Subsequently, 483 (237 males and 246 females) were screened for LTBI, including the administration of a two-step TST and chest X-ray. Of these patients, 15 had a clinical diagnosis of LTBI and 8 were given prophylactic TB treatment (Fig. 2). The average age was 62.3 years (standard deviation 16.3) and 59 % of patients were white, while 41 % were non-white.

Table 1 shows the association between the TST results and the collected demographic factors when considering 5- and 10-mm induration cutoffs. When considering a 5-mm induration cutoff, there was no correlation between the TST results and age, sex, or dialysis site. Participants with a positive TST exhibited a statistical trend towards a higher rate of BCG vaccination than patients who tested negative (34 vs. 25 %, *p* = 0.10). Excluding patients with unknown BCG status from the analysis, BCG vaccination status become associated with a positive TST result (50 vs. 35 %; *p* = 0.05). Those with a negative TST were more likely to be white (64 %) than those who tested positive (29 %) (*p* < 0.01). There was no statistically significant difference between those who were on peritoneal dialysis (*n* = 101) versus those on facility-based hemodialysis (*n* = 382) (*p* = 0.75 at a 5-mm induration and *p* = 0.19 at a 10-mm induration). We found similar results in the univariate analyses of other demographic factors when considering a cutoff induration of 10 mm.

**Table 1:** Baseline characteristics stratified by TST results

Characteristic	Positive TST, 5 mm	Negative TST, 5 mm	*P* value	Positive TST, 10 mm	Negative TST, 10 mm	*P* value
	*n* = 62	*n* = 421		*n* = 45	*n* = 438	
Age (years)	61.2 ± 14.4	62.5 ± 16.6	0.57	63.7 ± 13.6	62.2 ± 16.6	0.57
Race			<0.01			<0.01
White	28.6 %	64.1 %		28.6 %	62.8 %	
Non-white	71.4 %	35.9 %		71.4 %	37.2 %	
BCG			0.1			0.15
Vaccinated	33.9 %	25.4 %		35.6 %	25.6 %	
Not vaccinated	33.9 %	48.5 %		33.3 %	48.0 %	
Unknown	32.3 %	26.1 %		31.1 %	26.5 %	
Chest radiograph			0.82			0.57
Suggestive of TB	22.6 %	20.7 %		26.7 %	20.3 %	
Not suggestive of TB	72.6 %	75.8 %		68.9 %	76.0 %	
Not performed	4.8 %	3.6 %		4.4 %	3.7 %	
Dialysis Center (HD only)			0.18			0.11
HSC	58.0 %	43.9 %		44.0 %	61.5 %	
SOGH	18.0 %	25.2 %		25.2 %	15.4 %	
SBGH	24.0 %	30.9 %		30.8 %	23.0 %	
Peritoneal dialysis	19.4 %	21.1 %	0.75	13.3 %	21.7 %	0.19
Clinical diagnosis of LTBI (TB Registry)	16.1 %	1.2 %	<0.01	20.0 %	1.4 %	<0.01

Age presented as mean ± standard deviation, categorical variables presented as percentages

*TST* tuberculin skin test, *BCG* Bacillus Calmette-Guérin, *TB* tuberculosis, *HSC* Health Sciences Centre, *SOGH* Seven Oaks General Hospital, *SBGH* Saint Boniface General Hospital, *LTBI* latent tuberculosis infection

Based on a 5-mm threshold, 62 (13 %) of the 483 patients had a positive TST result, with 42 testing positive on the first administration and 20 testing positive on the second administration. Of the patients who tested positive, 14 had evidence of LTBI on X-ray, yielding a sensitivity of 14 %. Of patients testing negative, 319 had no radiographic evidence of LTBI, giving a specificity of 88 %. When a 10-mm induration was considered, 45 (9 %) patients tested positive, with 28 testing positive on the first administration and 17 testing positive on the second administration. Of these, 12 showed radiographic evidence of LTBI, resulting in a sensitivity of 12 % and a specificity of 91 %. Finally, when using multiple risk factors including radiographic evidence of disease, history of close contact with TB infection, or previous history of disease as a the reference standard for LTBI in a secondary analysis, the sensitivity and specificity of the TST based on a 5-mm induration was 15 and 88 %, respectively (contingency tables are available in Additional file 1).

### Association of TST results with prophylaxis

Linking the study cohort to provincial TB and LTBI registries, we found that only 8 patients received LTTB prophylaxis. Their mean age was 54.3 years, and the age of those who did not fit the criteria for prophylaxis was 62.5. Notably, among those patients who received prophylaxis, only 2 of the 8 had an abnormal chest radiograph. Due to the small number of individuals who received prophylaxis for LTBI, we lacked statistical power to compare the group with those who received no prophylaxis. An overview of the characteristics of patients who received isoniazid (INH) or rifampin (RMP) is displayed in Table 2.

**Table 2:** Baseline characteristics of patients treated for LTBI

Characteristic	*N* = 8
Age (years)	54.3 ± 14.7
BCG, *n* (%)	
Vaccinated	5 (62.5 %)
Not vaccinated	2 (25.0 %)
Unknown	1 (12.5 %)
Chest radiograph	
Suggestive of TB	2 (25 %)
Not suggestive of TB	6 (75 %)
Dialysis Center (HD only)	
HSC	5 (71 %)
SOGH	1(14 %)
SBGH	1 (14 %)
Peritoneal dialysis	1 (13 %)
Clinical diagnosis of LTBI, *n* (%)	8 (100 %)

*LTBI* latent tuberculosis infection, *BCG* Bacillus Calmette-Guérin, *TB* tuberculosis, *HSC* Health Sciences Centre, *SOGH* Seven Oaks General Hospital, *SBGH* Saint Boniface General Hospital

At the time of data linkage, we noted that 3 patients who were part of the 2008 screening protocol subsequently developed active TB infection. All three were Canadian-born non-white females who displayed no symptoms of TB infection at the time of TST screening. The screening protocol demonstrated heterogeneous results across chest radiographs and TST tests for these 3 patients.

## Discussion

In our study examining the diagnostic accuracy of the TST for detecting LTBI in a Canadian dialysis population, we found that the TST had poor sensitivity at both 5- and 10-mm thresholds of induration. Only 10 of the 62 patients who had a positive TST (and 5 patients with a negative TST) were found to have clinically diagnosed LTBI in the Manitoba TB and LTBI registries. Furthermore, only 8 of the 15 patients with LTBI were prescribed prophylaxis. In addition, one of three active TB cases had both a normal chest X-ray and negative TST result.

We hypothesize that the diminished sensitivity of the TST is likely a result of high rates of cutaneous anergy (40–50 %) reported in those with kidney failure [9, 11, 21]. Furthermore, false-positive results often occur in patients exposed to non-tuberculosis mycobacteria and the BCG vaccine [7, 11, 16, 17], also in concordance with our findings.

There are four drugs commonly used to treat tuberculosis: INH, RMP, pyrazinamide (PZA), and ethambutol (EMB), with INH being the first-line prophylaxis. The use of INH is not benign; there are many possible adverse effects including hepatitis, skin rash, flu-like syndrome, thrombocytopenia, and gastrointestinal upset. The incidence of adverse events from INH increases with age and is greatest in patients over 65 with comorbid conditions [22]. This may explain the small number of patients who were offered LTBI prophylaxis, as many patients may have been ineligible due to advanced age and comorbidities such as liver disease. Moreover, standard of care requires transplant candidates, who are often younger [23, 24], to receive the TST and prophylaxis if needed. This may have further contributed to the selection of younger patients offered pharmacologic therapy. Finally, these considerations highlight the importance of selectively screening only those patients who will actually qualify for prophylaxis, as recommended by the Canadian Tuberculosis Standards [22].

Our results are in keeping with previous findings from developing countries and highlight the low sensitivity and specificity of the TST in diagnosing LTBI in those with kidney failure [11, 16, 21], as well as the low rates of corresponding prophylaxis in those who do test positive with the TST [8, 25, 26]. A significant association was found between TST results and race, with white participants being less likely to test positive for LTBI than those from other ethnicities. This is not surprising because while overall TB rates in Canada have consistently declined, TB rates for Canadian-born aboriginal and foreign-born individuals are rising [22]. A similar trend has been noted in the USA with both overall and foreign-born TB rates declining but foreign-born rates decreasing at a much slower rate. Hispanic and Asian US immigrants may be affected up to 26 times more than non-Hispanic white individuals [27]. This phenomenon is likely due to factors such as immigration from endemic TB regions, generally lower socioeconomic status and educational resources, and more crowded living conditions among non-whites [28, 29].

While the TST is currently the most widely used diagnostic tool for LTBI, our results support the investigation of alternative diagnostic strategies for LTBI in dialysis patients. Several bodies now recommend using interferon-gamma release assays (IGRAs) as an alternative to TST in order to screen at-risk populations in high- and middle-income countries, including patients on dialysis and those awaiting transplant [22, 30]. IGRAs have increased sensitivity and specificity in dialysis patients; however, they are not typically employed in kidney failure patients [31], presumably because of the increased cost on a per-test basis [32].

IGRAs have many advantages over the TST as a diagnostic tool for LTBI. Like the TST, IGRAs assess the immune response to TB antigens, but with increased specificity [33], and although IGRAs are also subject to anergy [34], they may be less susceptible to uremic immunosuppression [33, 35]. Furthermore, the “boosting” effect that can been seen in patients subjected to repeated TST testing is absent [33, 35]; they require less technical acumen, eliminating diagnostic variability, and they are less cumbersome for the patient since a follow-up assessment is not needed. While the immediate costs of IGRAs are higher than those of TST, they may be more cost-effective in this population when considering long-term outcomes [32]. Some studies have shown that the sensitivity of IGRAs may be reduced post hemodialysis, but even under these conditions, IGRAs have a higher sensitivity than the TST [35, 36].

Our study has important clinical and research implications. First, since the TST has poor diagnostic performance for diagnosis of LTBI in those with kidney failure, it must be cautiously interpreted as a screening tool in dialysis patients. Alternatives such as IGRAs may perform better, but this needs to be confirmed in future studies in dialysis patients. Finally, screening for LTBI should only be performed in patients for whom the risk-to-benefit ratio of prophylaxis is favorable. Generally speaking, this would include younger patients and those waitlisted for kidney transplant, excluding the most elderly and highly comorbid patients for whom the risk of INH therapy may exceed the benefit of eradicating LTBI.

Our study has several strengths. We were able to provide a sample size of 483 patients who received a two-step TST, to date, one of the largest studies in a dialysis population from a non-endemic location [12, 37]. Through administrative database linkages, we were able to capture data informing which patients subsequently received pharmacological intervention. These data linkages enabled us to assess the clinical utility of the TST over a 5-year follow-up period.

There are also important limitations to our analysis. First, due to the lack of a true gold standard for diagnosis of LTBI [12, 19], we used radiographic evidence of previous TB infection or radiographic evidence plus risk of exposure and history of infection as the reference standard in our primary and secondary analyses, respectively. Although these criteria are known to be flawed reference standards, they have been widely applied in the published literature [12, 37], thereby facilitating comparisons with other studies and increasing the generalizability of our findings. Additionally, many of the risk assessment variables captured in our survey were self-reported and subject to patient recall bias. Many of these risk variables were also incompletely reported, as was the case for BCG vaccination status. Finally, due to the low number of patients who reported prior contact or history of TB infection, and their self-reported nature, we could not meaningfully analyze the sensitivity and specificity of these variables in isolation.

## Conclusions

Despite its continued use in the clinical setting, the TST has poor diagnostic accuracy and clinical utility for LTBI screening in patients on dialysis. The poor correlation between TST results, currently used proxies for LTBI, and prophylaxis rates indicates a need for a better gold standard definition of LTBI. Finally, further study is recommended to determine the diagnostic accuracy and cost utility of IGRAs in those with kidney failure and other immunocompromised populations.

## Additional file

Additional file 1: Contingency tables. 2 x 2 table describing the number of patients in each test category for the tuberculin skin test and chest x ray. (XLS 24 kb)

## Abbreviations

BCG: Bacillus Calmette-Guérin
HIPC: Health Information Privacy Committee
HIV: human immunodeficiency virus
IGRA: interferon-gamma release assay
INH: isoniazid
iPHIS: Integrated Public Health Information System
KDIGO: Kidney Disease: Improving Global Outcomes
LTBI: latent tuberculosis infection
MH: Manitoba Health
MRP: Manitoba Renal Program
RMP: rifampin
TB: tuberculosis
TST: tuberculin skin test
WHO: World Health Organization
WRHA: Winnipeg Regional Health Authority
